# Report on Cholera, by the Sanitory Committee of Board of Health of New York

**Published:** 1849

**Authors:** 


### ARTICLE II.

*Report of the Standing Sanatory Committee of the Board of Health of the City of New York, on the subject of Asiatic Cholera, at present prevailing at the Quarantine Establishment of New York, at Staten Island.* pp. 24, octavo.

This is a report of the cholera as it was recently prevailing at New York, and shows the manner of its commencement on board the ship New York seven days previous to her arrival at quarantine. The cases during this week were seven, all of which proved fatal. The vessel lay without landing her passengers from Friday night until Sunday noon, during which time there occurred twelve new cases.

There appears to have been used every precaution to prevent the spread of the disease.

The symptoms of the disease, as it prevailed for about two weeks after the landing, were those that our readers are familiar with as those of Asiatic Cholera. Dr. Whiting, the author of the report, and health officer at quarantine, says :

In this disease there has been but one stage—that of collapse—although every pains have been taken to detect the first deviations from health, directions given to all to communicate them at once, and persons employed to inspect them constantly, and a physician to pass among them at all hours of the night and day. The first intimations are the extreme symptoms, defying the most prompt and decided remedies.

In reference to the cause of the disease, there seems to be nothing more intelligent than in reference to cholera generally heretofore ; the report says on this head :

The only one that may be adduced, is the depressing influence of grief at being driven from their homes and flourishing trades ; but this is not apparent in their appearance or manner. They enjoyed promiscuously with the other passengers, the best accommodations, and I am assured by Capt. Lines, that their fare was the same with the other passengers.

I have examined their provisions on board ship, casks of bacon, rice, flour, beans, biscuit and potatoes, unheaded and exhibited to me, as it has been dealt to them, and I am sure that more wholesome or sweeter provisions could not be provided.

They are all healthy and robust—have not been exposed to the cholera at home, and have since leaving their port of departure, shared equally with the exempt, the comforts and privations of a sea voyage, variations of wind and weather, have breathed the same air, and fed on the same food.

In reference to treatment, it certainly cannot be regarded as very flattering; as about one half of the cases attacked died: that is, of sixty three cases, twenty-nine died. There is, however, a great difference between the results of the first and last cases. Of the first thirty cases, twenty died; while of the thirty-three occurring after the 11th of December, only nine proved fatal, owing to the improvement in the plan of treatment, or change in the character of the disease. As the remarks upon treatment are of more importance, bearing upon the course to be pursued should it prevail, we quote the conclusions to which the Doctor comes, without comment:

From the results of the first thirty cases, and post mortem revelations, I became convinced that the stimulating plan was not the treatment for *this* cholera, and abandoned at first the mustard, then the capsicum, ammonia, brandy, wine whey, etc., and relied on calomel in large doses, with opium, Dover's powder, and camphor.

With regard to camphor, even though it has been always lauded, and by some as *the specific* in cholera, I entertain suspicions of its utility.

The treatment I have now adopted and adhere to, from its decided agency in controlling the symptoms and inducing early reaction, is the exhibition of moderate doses of calomel, with morphine, at short intervals. Five grains of calomel, with a quarter of a grain of sulph. morphia, is at first given to an adult; in a half of an hour, or one hour, a scruple dose of calomel is exhibited, and is usually retained; afterward, a pill of Cal. grs. v, Sulph Morphine gr $\frac{1}{4}$, is given each hour, two hours or three hours, as the effect may indicate. This is observed in the subsidence of the pain and spasms, the diminished quantity and frequency of the evacuations, the return of warmth, and the restoration of the pulse.

This treatment is continued until some indications of bilious action appear; the first is usually a change of color and consistence from the light, thin, rice water, to a greenish, and then brown or brownish yellow color. The evacuations from the stomach and bowels will frequently continue green, or of the color of sulphate of copper, for hours, but I have not known a single case to relapse where this effect had once been produced.

I was led to substitute the morphine for opium, from its being less liable to disturb the stomach or to produce narcosis, an effect to be deprecated in this stage of congestion, except it result naturally from the obviation of pain and excitement.

In children, however, under six or seven years, I have used Dover's powder in preference to morphine, as being more manageable in regard to the dose. A very simple remedy, but one that I have used in children with happy effects, has been the tea of the spearmint, given hot in the first stages, and afterwards cold, in a small quantity, a large spoonful occasionally.

The most valuable external means is the stream of hot vapor of alcohol, poured over the patient by a very simple apparatus at the foot of the bed. This is a large alcohol lamp placed under a sheet iron cylinder, with a pipe running from it. The lamp is placed on the floor, and the tube with an elbow, and terminating in a large funnel to elevate the clothes.

This and hot mustard applications are the only external means that I rely on. They are potent, and can be continued without the fatigue or exposure of the patient, a paramount desideratum, as there is plenty of both to contend with as the inevitable effects of the disease.

---

